# Do hypokyphotic adolescent idiopathic scoliosis patients treated with Ponte osteotomy obtain a better clinical efficacy? A preliminary retrospective study

**DOI:** 10.1186/s13018-022-03390-0

**Published:** 2022-11-16

**Authors:** Fei Wang, Kai Chen, Tao Ji, Yuegang Ma, Hao Huang, Ping Zhou, Xianzhao Wei, Ziqiang Chen, Yushu Bai

**Affiliations:** 1 Department of Orthopedic Surgery, Shaoxing People’s Hospital, Shaoxing, Zhejiang Province China; 2 Department of Orthopedics, Shanghai Changhai Hospital, Shanghai, 200433 China; 3 Department of Educational Administration, Naval Medical University, Shanghai, 200433 China

**Keywords:** Adolescent idiopathic scoliosis, Ponte osteotomy, Hypokyphotic curve

## Abstract

**Study design:**

A retrospective case–control study.

**Objective:**

To evaluate whether Ponte osteotomy improves thoracic kyphosis and to determine its clinical efficacy in hypokyphotic adolescent idiopathic scoliosis (AIS).

**Methods:**

Eighty consecutive Lenke type 1 AIS patients with hypokyphotic curves who underwent posterior spinal fusion by one spine surgeon at a single institution were recruited. According to whether Ponte osteotomy was performed, the patients were divided into two groups. The preoperative, immediate, one-year postoperative, and two-year postoperative radiographs were analyzed. The demographic characteristics, surgical information, radiographic parameters, Scoliosis Research Societye-22 (SRS-22) questionnaire, and complications were compared.

**Results:**

The sagittal alignment and coronal alignment were both improved in the Ponte group and the control group postoperatively. There was no significant difference in the preoperative parameters between the two groups, except the TL/L, CB, and LL. Significant differences were found in the MT (15.18° ± 2.84° vs. 20.33° ± 3.75°, *P* < 0.001) and TK (24.23° ± 2.71° vs. 19.93° ± 2.38°, *P* < 0.001) at the two-year follow-up. The Ponte group had a longer operation time and more intraoperative blood loss. No significant difference was observed between the groups in the SRS-22 scores at the final follow-up.

**Conclusions:**

Ponte osteotomy could obtain better coronal correction and sagittal contour restoration in AIS patients with hypokyphosis. However, Ponte osteotomies might lead to more intraoperative blood loss and longer operation time. Moreover, no discrepancy was found in the postoperative health-related quality of life of the included patients. Therefore, we considered that the Ponte osteotomy may be an alternative method to restore the desired thoracic kyphosis, which needs further study.

## Introduction

The main surgical purpose of adolescent idiopathic scoliosis (AIS) includes the correction of spinal deformities, prevention of a progressive curvature, and improvement of appearance deformities. In the past, a majority of scholars evaluated the coronal correction to assess the surgical outcomes. However, an increasing number of spinal surgeons have begun to focus on the influence of the sagittal alignment on the surgical outcomes in recent years. Abnormal sagittal sequences may result in failure of internal instrumentation and unsatisfactory clinical efficacy [1]. Moreover, the restoration of sagittal alignment has been directly related to the improvement of pain and function after spinal deformity surgery.

Posterior pedicle screw instrumentation and correction have gained popularity in the treatment of AIS for more than three decades. Several studies have shown that patients with thoracic pedicle screws obtained better coronal and transverse plane corrections (save the number of fusion levels) and decreased the intraoperative blood loss compared with hook or hybrid instrumentation [2–4]. However, quite a few studies have reported a loss of thoracic kyphosis may occur after pedicle screw fixation [5, 6]. More importantly, the sagittal alignment, such as thoracic kyphosis, could be correlated with the improvement of health-related quality of life (HRQoL) in adolescent and adult idiopathic scoliosis patients [7–9]. Therefore, the restoration of normal thoracic kyphosis plays a vital role in evaluating the surgical outcomes, and various scholars have proposed different techniques to restore normal thoracic kyphosis, such as simultaneous translation on two rods, cantilever reduction, the universal clamp hybrid system, sublaminar bands, and Ponte osteotomy [10–14]. Ponte osteotomy, as a surgical technique to restore a normal thoracic kyphosis, has been a relatively popular in recent years.

However, there are some controversies among scholars about whether Ponte osteotomy improves the coronal correction and restores the sagittal alignment. To date, no comparative study has been performed whether Ponte osteotomy with pedicle screw constructs benefits better in hypokyphotic deformities. Therefore, the purpose of this study was to explore whether the adoption of a Ponte osteotomy could restore a normal thoracic kyphosis in hypokyphotic patients with Lenke type 1 AIS and obtain better clinical efficacy.

## Methods

### Setting and patient population

Eighty consecutive patients with Lenke type 1 AIS who were treated with one-stage posterior thoracic curve pedicle screw instrumentation and obtained a correction between January 2011 and January 2017 were enrolled in this study. The inclusion criteria were as follows: (1) a diagnosis of AIS and aged between 10 and 18 years; (2) a main thoracic curve > 40°; (3) no previous flexibility-modifying surgery; (4) a thoracic kyphosis curve < 10°; and (5) a follow-up period of more than two years after the operation. According to whether Ponte osteotomy was performed, 40 patients were included in the Ponte group, and the other patients were included in the control group. The study was approved by the Institutional Review Board of Shanghai Changhai Hospital (CHEC20170183), and all participants in our study provided written informed consent for the study.

### Surgical technique

**Fig. 1:**
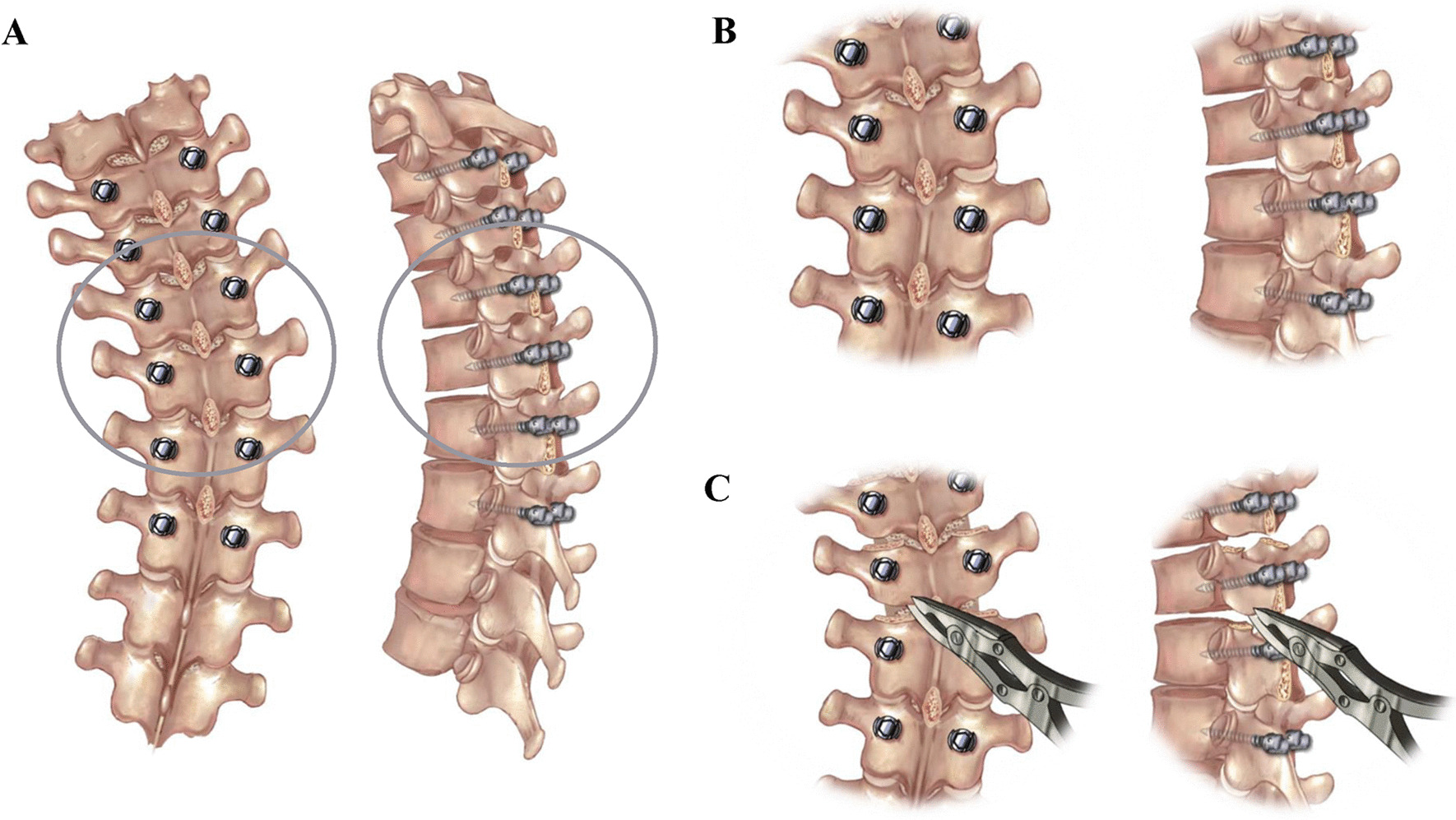
The illustration of management on the apical vertebrae area. **A** The posterior–anterior (PA) and lateral overview of surgical site, and the region of grew circle correlated with apex region; **B** The local posterior–anterior and lateral view on the apical vertebrae area in the control group, where only spinous process was resected; **C** The local posterior–anterior and lateral view on the apical vertebrae area in the Ponte group, where apart from spinous process, superior and inferior articular processes, part of vertebral lamina and ligamentum flavum were resected

The AIS patients were placed in the prone position on a Jackson radiolucent spinal table after the induction of general anesthesia. The surgeon made a posterior midline incision and dissected the paraspinal muscle to the tip of the transverse process for all the levels of spinal fusion. Pedicle screws were inserted bilaterally with a free-hand technique [15]. Then, the spinous processes of the operative segments were removed. In the Ponte group, the Ponte osteotomies (resection of superior and inferior articular processes, part of vertebral lamina and ligamentum flavum) were additionally performed in the apex vertebrae area as described by Ponte et al. [16], and the number of detailed osteotomy segments was an average of three segments according to individual’s situation. The management on the apical vertebrae area in the two groups is shown in Fig. 1. Based on the physiological curvature of the human spine, a concave rod was first placed at the distal and proximal foundations. The curve correction was achieved using a lifting tool from both ends to the middle, followed by slight concave distraction and convex compression. A rod rotation was performed. During surgery, the somatosensory-evoked potentials were routinely determined for the intraoperative monitoring of spinal cord function. We used pedicle screws with diameters between 5 and 6 mm and lengths ranging from 30 to 50 mm. The rod material (Expedium, Depuy Synthes, USA) was CoCr alloy and its diameter was 5.5 mm. There were no deviations from the preoperative designed surgical strategies. All operations were performed by one single experienced spinal surgeon (YS. B.) using the same operative technique.

### Radiographic and clinical assessment

Preoperative, immediate postoperative (i.e., the 1st week), three-month, one-year, and two-year follow-up radiographs were obtained on long cassettes by certified radiology technicians in a standardized fashion. The parameters measured on the coronal radiographs were as follows: (1) the Cobb angle of proximal thoracic (PT), main thoracic (MT), and thoracolumbar/lumbar (TL/L) curve, (2) coronal balance (CB), and (3) radiographic shoulder height (RSH). CB was defined as the horizontal distance between the center sacral vertical line (CSVL) and the coronal C7 plumb line (C7PL). A negative value indicated that the C7 plumb line was right to the CSVL. RSH was the perpendicular distance in the soft tissue shadows that were directly superior to the acromioclavicular joint. A negative value indicated that the right shoulder was elevated. Four sagittal radiographic parameters were measured: (1) the cervical sagittal alignment (CSA), (2) thoracic kyphosis (TK), (3) lumbar lordosis (LL), and (4) sagittal vertical axis (SVA). The C2-C7 lordosis was used as the current parameter for the CSA, which was measured from the inferior end plate of C2 to the inferior end plate of C7. The TK was the angle between the lines that were drawn from the T4 superior end plate and T12 inferior end plate. The LL was the angle between lines drawn from the L1 superior end plate and L5 inferior end plate. A negative value indicated lordosis on the sagittal plane. The SVA was the distance between the posterosuperior point of the sacral plate and the lateral C7 plumb line. A negative value indicated that the C7 plumb line was posterior to the sacrum posterior corner. The flexibility rate of main thoracic curve was calculated according to bending X-ray.

The operative time (OT), intraoperative blood loss (IBL), and number of pedicle screws (NPS) were recorded. The Scoliosis Research Society (SRS)-22 questionnaire was applied to assess the clinical outcomes of the AIS patients both preoperatively and at the two-year follow-up.

The radiographs were measured by two authors (F.W. and K.C.) of this study, who performed the measurements independently. An experienced spinal surgeon (ZQ. C.) reviewed the medical records and plain radiographs of all patients.

### Statistical analyses

Statistical analyses were performed utilizing SPSS statistical software 21.0 (Chicago, IL, USA). The preoperative and postoperative radiographic parameters were compared using paired t tests and Mann–Whitney U tests when appropriate. Comparisons between the two groups were performed using a single factor ANOVA test for continuous variables. *P* < 0.05 was considered statistically significant.

## Results

### Patients demographics

The Ponte osteotomy group consisted of 40 patients with AIS, including 36 females (90%) and 4 males (10%). The average age at surgery was 14.50 ± 1.77 years old. The average Risser grade was 3.80 ± 0.82. The mean operation time was 262.00 ± 28.80 min. There were 26 patients with lumbar modifier A, 10 with lumbar modifier B, and 4 with lumbar modifier C. The upper instrumented vertebrae (UIV) located at T3 in 6 patients, T4 in 19 patients, and T5 in 15 patients. Meanwhile, the lower instrumented vertebrae (LIV) located at T12 in 17 patients and L1 in 23 patients. The average number of Ponte osteotomies performed was 3.25 ± 0.44, and the mean number of levels fused was 9.53 ± 0.51. The mean intraoperative blood loss was 1103.25 ± 115.06 ml (as shown in Tables 1 and 2).

**Table 1:** Preoperative parameters comparison between Ponte group and control group

Parameters	Control group	Ponte group	*P* value
Age (year)	15.13 ± 1.57	14.50 ± 1.77	0.099
Gender			0.675
Male	2	4	
Female	38	36	
Risser sign	4.00 ± 0.85	3.80 ± 0.82	0.287
Lumbar modifier			0.198
A	18	26	
B	16	10	
C	6	4	
PT (°)	27.00 ± 3.70	25.55 ± 3.69	0.083
MT (°)	50.03 ± 4.88	48.10 ± 3.93	0.056
TL/L (°)	26.93 ± 3.23	22.40 ± 4.32	**0.004**
CB (mm)	− 3.85 ± 15.24	− 13.20 ± 13.73	**0.005**
RSH (mm)	− 5.58 ± 12.47	− 7.23 ± 12.45	0.555
FR (%)	53.66 ± 8.34	56.75 ± 7.56	0.108
CSA (°)	3.08 ± 9.77	4.48 ± 6.80	0.459
TK (°)	6.45 ± 2.95	5.30 ± 3.18	0.098
LL (°)	− 39.58 ± 5.11	− 36.15 ± 4.44	** < 0.001**
SVA (mm)	− 17.18 ± 12.22	− 16.20 ± 11.42	0.446

*PT* proximal thoracic, *MT* main thoracic, *TL/L* thoracolumbar/lumbar, *CB* coronal balance, *RSH* radiographic shoulder height, *FR* flexibility rate, *CSA* cervical sagittal alignment, *TK* thoracic kyphosis, *LL* lumbar lordosis, *SVA* sagittal vertical axis

Statistically significant values were bolded

**Table 2:** Postoperative 2-year follow-up parameters comparison between Ponte group and control group

Parameters	Control group	Ponte group	*P* value
PT (°)	15.25 ± 2.17	14.43 ± 2.58	0.126
MT (°)	20.33 ± 3.75	15.18 ± 2.84	** < 0.001**
TL/L (°)	8.38 ± 2.92	7.60 ± 2.23	0.187
CB (mm)	− 5.95 ± 9.57	− 9.18 ± 9.48	0.134
RSH (mm)	− 3.10 ± 7.76	− 4.53 ± 7.19	0.379
CSA (°)	0.40 ± 8.96	1.08 ± 6.74	0.704
TK (°)	19.93 ± 2.38	24.23 ± 2.71	** < 0.001**
LL (°)	− 40.25 ± 5.92	− 42.98 ± 5.21	0.690
SVA (mm)	− 10.18 ± 9.54	− 7.93 ± 10.08	0.292
OT (min)	229.50 ± 26.84	262.00 ± 28.80	** < 0.001**
IBL (ml)	979.75 ± 171.71	1103.25 ± 115.06	** < 0.001**
NPS	13.53 ± 1.13	13.63 ± 0.84	0.655
Fusion segment	9.45 ± 0.50	9.53 ± 0.51	0.508
UIV (level)			0.630
T3	4	6	
T4	23	19	
T5	13	15	
LIV (level)			0.459
T12	15	17	
L1	25	23	

*PT* proximal thoracic, *MT* main thoracic, *TL/L* thoracolumbar/lumbar, *CB* coronal balance, *RSH* radiographic shoulder height, *CSA* cervical sagittal alignment, *TK* thoracic kyphosis, *LL* lumbar lordosis, *SVA* sagittal vertical axis, *OT* operative time, *IBL* intraoperative blood loss, *NPS* number of pedicle screw, *UIV* upper instrumented vertebrae, *LIV* lower instrumented vertebrae

Statistically significant values were bolded

The control group also consisted of 40 patients with AIS, and this group included 38 females (95%) and 2 males (5%). The average age at surgery was 15.13 ± 1.57 years old. The average Risser grade was 4.00 ± 0.85. The mean operating time was 229.50 ± 26.84 min. There were 18 patients with a lumbar modifier A, 16 with a lumbar modifier B, and 6 with a lumbar modifier C. The UIV was located at T3 in 4 patients, T4 in 23 patients, and T5 in 13 patients. Meanwhile, the LIV was located at T12 in 15 patients and L1 in 25 patients. The mean intraoperative blood loss was 979.75 ± 171.71 ml, and the mean number of levels fused was 9.45 ± 0.50 (as shown in Tables 1 and 2).

### Radiographic analysis

In the Ponte group, there were no significant differences between the preoperative and immediate postoperative CSA, between the immediate postoperative and three-month follow-up PT, TL/L, CSA, and LL, between the three-month and one-year follow-up RSH, TK, and SVA, between the one-year and two-year follow-up PT, MT, CB, RSH, CSA, TK, LL, and SVA. There were significant differences between the preoperative and immediate postoperative PT, MT, TL/L, CB, RSH, TK, LL, and SVA (*P* < 0.001, *P* < 0.001, *P* < 0.001, *P* = 0.002, *P* = 0.004, *P* < 0.001, *P* < 0.001, and *P* = 0.006, respectively), the immediate postoperative and three-month follow-up MT, CB, RSH, TK, and SVA (*P* = 0.012, *P* < 0.001, *P* < 0.001, *P* = 0.013, and *P* < 0.001, respectively), the three-month and one-year postoperative PT, MT, TL/L, CB, CSA, and LL (*P* = 0.004, *P* < 0.001, *P* < 0.001, *P *= 0.006, *P* = 0.020, and *P* = 0.009, respectively), and the one-year and two-year postoperative TL/L (*P* < 0.001). (Table 3).

**Table 3:** Radiographic parameters comparison among preoperative and postoperative follow-up in Ponte Group

Parameters	Preoperative	Immediate postoperative	3 month postoperative	1 year postoperative	2 year postoperative	Pre versus Im-post	Im-post versus 3 m-post	3 m-post versus 1y-post	1y-post versus 2y-post
PT (°)	25.55 ± 3.69	12.98 ± 2.71	13.18 ± 2.58	14.08 ± 2.30	14.43 ± 2.58	** < 0.001**	0.518	**0.004**	0.289
MT (°)	48.10 ± 3.93	13.90 ± 3.23	13.00 ± 2.61	14.48 ± 3.20	15.18 ± 2.84	** < 0.001**	**0.012**	** < 0.001**	0.083
TL/L (°)	22.40 ± 4.32	8.03 ± 4.01	8.53 ± 2.97	9.53 ± 2.68	7.60 ± 2.23	** < 0.001**	0.215	** < 0.001**	** < 0.001**
CB (mm)	− 13.20 ± 13.73	− 19.10 ± 12.48	− 11.48 ± 8.53	− 8.80 ± 7.77	− 9.18 ± 9.48	**0.002**	** < 0.001**	**0.006**	0.756
RSH (mm)	− 7.23 ± 12.45	− 13.03 ± 11.18	− 5.10 ± 8.00	− 4.85 ± 6.78	− 4.53 ± 7.19	**0.004**	** < 0.001**	0.677	0.638
CSA (°)	4.48 ± 6.80	5.08 ± 6.36	3.93 ± 7.04	2.23 ± 7.64	1.08 ± 6.74	0.507	0.125	**0.020**	0.081
TK (°)	5.30 ± 3.18	21.68 ± 3.62	23.38 ± 3.20	24.00 ± 3.52	24.23 ± 2.71	** < 0.001**	**0.013**	0.191	0.618
LL (°)	− 36.15 ± 4.44	− 40.08 ± 5.21	− 40.95 ± 3.93	− 42.38 ± 3.51	− 42.98 ± 5.21	** < 0.001**	0.105	**0.009**	0.132
SVA (mm)	− 16.20 ± 11.42	− 22.95 ± 8.72	− 12.18 ± 9.39	− 6.40 ± 9.71	− 7.93 ± 10.08	**0.006**	** < 0.001**	0.052	0.447

*PT* proximal thoracic, *MT* main thoracic, *TL/L* thoracolumbar/lumbar, *CB* coronal balance, *RSH* radiographic shoulder height, *CSA* cervical sagittal alignment, *TK* thoracic kyphosis, *LL* lumbar lordosis, *SVA* sagittal vertical axis

Statistically significant values were bolded

In the control group, there were no significant differences between the preoperative and immediate postoperative RSH, CSA, and SVA, between the immediate postoperative and three-month follow-up PT, MT, RSH, and LL, between the three-month and one-year follow-up PT, MT, CB, RSH, TK, LL, and SVA, between the one-year and two-year follow-up PT, MT, TL/L, CB, RSH, CSA, TK, LL, and SVA. There were significant differences between the preoperative and immediate postoperative PT, MT, TL/L, CB, TK, and LL (*P* < 0.001, *P* < 0.001, *P* < 0.001, *P* = 0.001, *P* < 0.001, and *P* < 0.001, respectively), the immediate postoperative and three-month follow-up TL/L, CB, CSA, TK, and SVA (*P* = 0.007, *P* = 0.009, *P* = 0.040, *P* = 0.044, and *P* < 0.001, respectively), and the three-month and one-year postoperative TL/L and CSA (*P* = 0.017 and *P* = 0.048, respectively). (Table 4).

**Table 4:** Radiographic parameters comparison among preoperative and postoperative follow-up in control group

Parameters	Preoperative	Immediate postoperative	3 month postoperative	1 year postoperative	2 year postoperative	Pre versus Im-post	Im-post versus 3 m-post	3 m-post versus 1y-post	1y-post versus 2y-post
PT (°)	27.00 ± 3.70	14.10 ± 2.72	14.28 ± 2.41	14.80 ± 2.29	15.25 ± 2.17	** < 0.001**	0.636	0.136	0.155
MT (°)	50.03 ± 4.88	18.83 ± 4.01	19.38 ± 3.51	19.88 ± 3.08	20.33 ± 3.75	** < 0.001**	0.086	0.074	0.186
TL/L (°)	26.93 ± 3.23	12.55 ± 2.87	10.30 ± 2.86	8.93 ± 3.09	8.38 ± 2.92	** < 0.001**	**0.007**	**0.017**	0.083
CB (mm)	− 3.85 ± 15.24	− 9.90 ± 17.23	− 5.78 ± 10.92	− 5.33 ± 9.76	− 5.95 ± 9.57	**0.001**	**0.009**	0.473	0.259
RSH (mm)	− 5.58 ± 12.47	− 4.48 ± 13.08	− 2.95 ± 9.26	− 2.85 ± 9.21	− 3.10 ± 7.76	0.456	0.218	0.847	0.609
CSA (°)	3.08 ± 9.77	2.58 ± 9.24	1.33 ± 8.68	0.15 ± 9.10	0.40 ± 8.96	0.323	**0.040**	**0.048**	0.598
TK (°)	6.45 ± 2.95	20.00 ± 2.84	19.18 ± 2.45	19.55 ± 2.20	19.93 ± 2.38	** < 0.001**	**0.044**	0.408	0.319
LL (°)	− 39.58 ± 5.11	− 39.48 ± 9.62	− 41.15 ± 7.90	− 43.03 ± 7.93	− 40.25 ± 8.92	** < 0.001**	0.590	0.376	0.577
SVA (mm)	− 17.18 ± 12.22	− 15.73 ± 10.11	− 11.15 ± 8.41	− 9.43 ± 6.90	− 10.18 ± 9.54	0.680	** < 0.001**	0.124	0.259

*PT* proximal thoracic, *MT* main thoracic, *TL/L* thoracolumbar/lumbar, *CB* coronal balance, *RSH* radiographic shoulder height, *CSA* cervical sagittal alignment, *TK* thoracic kyphosis, *LL* lumbar lordosis, *SVA* sagittal vertical axis

Statistically significant values were bolded

**Fig. 2:**
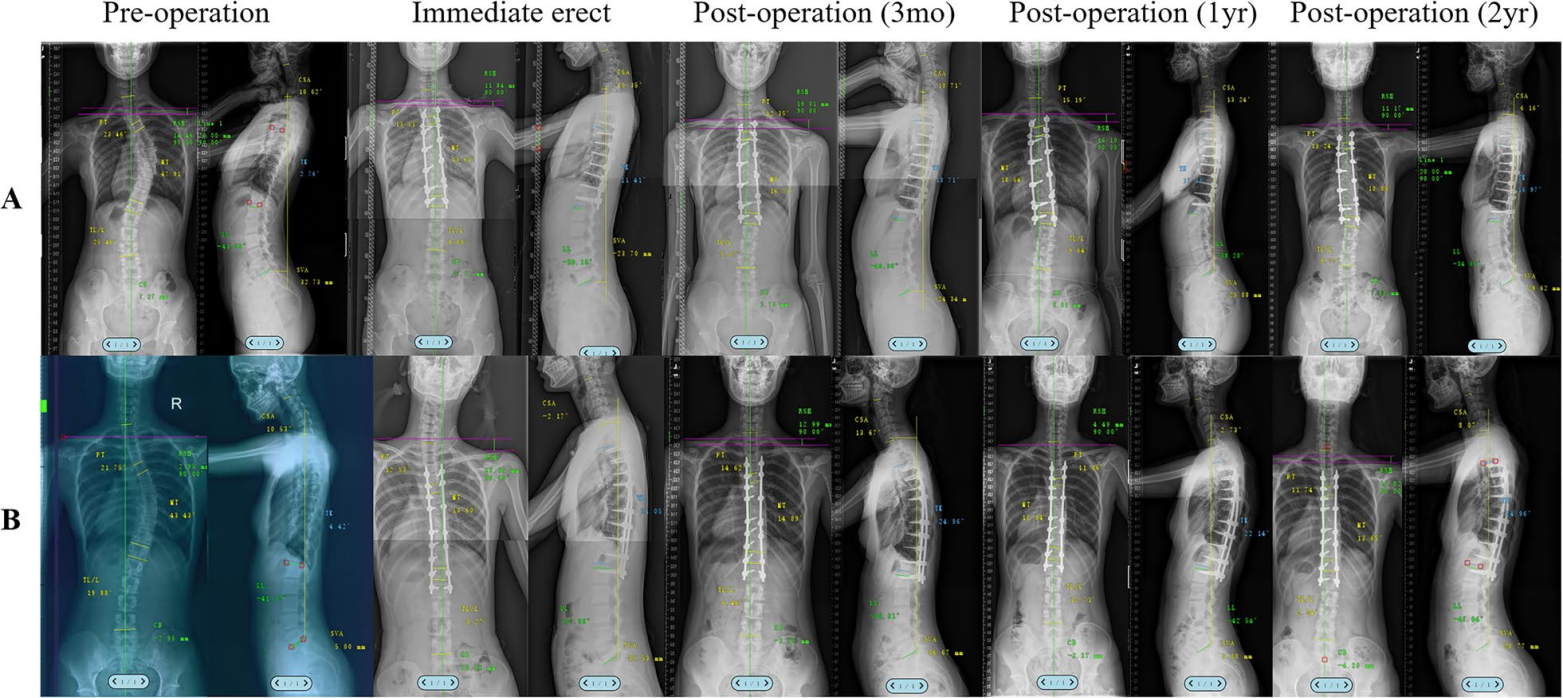
Typical cases of two groups in the follow-up. **A** A 16-year-old girl of Lenke type 1 AIS without Ponte osteotomy. Preoperative coronal Cobb angle of the PT, MT, and TL/L was 23.46°, 47.91°, and 25.46°, respectively. Preoperative sagittal Cobb angle of the CSA, TK, and LL was 10.62°, 2.76°, and − 41.83°, respectively. 2-year postoperative coronal Cobb angle of the PT, MT, and TL/L was 13.24°, 18.05°, and 9.77°, respectively. 2-year postoperative sagittal Cobb angle of the CSA, TK, and LL was 6.16°, 16.97°, and − 34.51°, respectively. **B** A 15-year-old girl of Lenke type 1 AIS with Ponte osteotomy. Preoperative coronal Cobb angle of the PT, MT, and TL/L was 21.75°, 43.43°, and 19.88°, respectively. Preoperative sagittal Cobb angle of the CSA, TK, and LL was 10.53°, 4.42°, and − 41.37°, respectively. 2-year postoperative coronal Cobb angle of the PT, MT, and TL/L was 11.74°, 13.65°, and 2.24°, respectively. 2-year postoperative sagittal Cobb angle of the CSA, TK, and LL was 8.07°, 24.96°, and − 47.04°, respectively

There were no significant differences in age, sex, Risser sign, PT, MT, RSH, FR, CSA, TK, or SVA between the two groups before the operation. At the two-year follow-up, there were significant differences in the MT, TK, OT, and IBL between the two groups. The typical cases are shown in Fig. 2.

### Clinical assessment

Preoperatively, patients in the Ponte osteotomy cohort had lower preoperative self-image and satisfaction scores than those in the control group. At the 2-year follow-up, there was no significant difference identified in any domain between the patients who underwent Ponte osteotomy and those who did not (Table 5).

**Table 5:** Comparison of preoperative and postoperative SRS-22 scores between Ponte group and control group

Domain	Preoperative			2-year postoperative follow-up		
	Control group	Ponte group	*P *value	Control group	Ponte group	*P* value
Pain	4.45 ± 0.50	4.50 ± 0.51	0.659	4.53 ± 0.51	4.50 ± 0.51	0.826
Self-image	3.78 ± 0.58	3.48 ± 0.55	**0.020**	4.20 ± 0.52	4.35 ± 0.48	0.184
Function	4.50 ± 0.51	4.48 ± 0.51	0.826	4.35 ± 0.58	4.40 ± 0.55	0.692
Mental health	4.13 ± 0.69	4.10 ± 0.55	0.857	4.20 ± 0.56	4.15 ± 0.66	0.717
Satisfaction	3.90 ± 0.59	3.63 ± 0.54	**0.033**	4.35 ± 0.53	4.50 ± 0.51	0.201
Total	4.02 ± 0.28	4.04 ± 0.26	0.051	4.33 ± 0.23	4.38 ± 0.25	0.314

Statistically significant values were bolded

### Complications

Two patients in the Ponte osteotomy group developed superficial incisional infections that required anti-infection treatment and incisional debridement. One patient in the control group had postoperative abdominal pain, abdominal distension, nausea, and vomiting and was treated with gastrointestinal decompression. There was no neurological complication occurrence in two groups.

## Discussion

Some authors considered that AIS patients with thoracic curve frequently presented with decreased thoracic kyphosis due to lordoscoliosis with a vertebral rotatory deformity [17]. A relative overgrowth of the anterior spinal column leading to hypokyphosis of the thoracic segments has been theorized as one of the etiologies of AIS [18]. This hypokyphosis not only affected the patients’ appearance and psychological function, but also the patient’s pulmonary function and sagittal alignment [19, 20]. Therefore, the restoration of a normal thoracic kyphosis is one of the most important factors in the evaluation of clinical efficacy. Recently, several investigators have found that Ponte osteotomy might improve the thoracic kyphosis restoration.

Shah et al. [14] analyzed the results of Ponte osteotomy for the correction of AIS patients with hypokyphosis and reported that with Ponte osteotomy, patients had a significant increase in the lateral thoracic kyphosis (T5–T12) from 8.1° to 18.3°. However, this study did not include a control group. Javier et al. [21] reported that Ponte osteotomy helped to achieve a normal sagittal profile, with an increase in thoracic kyphosis from 6° to 17°. Moreover, they found that there was a significant difference between the postoperative TK of 12.6° ± 4.3° in the control group and TK of 17° ± 3.2° in the Ponte group. In our study, we found that the mean TK increased from 5.3° to 24.23° at the final follow-up in the Ponte group. Moreover, Ponte osteotomy achieved better sagittal correction for thoracic kyphosis in AIS patients than those who did not apply Ponte osteotomy (24.23° ± 2.71° vs. 19.93° ± 2.38°, *P* < 0.001). Therefore, we believed that a Ponte osteotomy involving a posterior release and a posterior column lengthening could result in the postoperative restoration of a normal thoracic kyphosis in the hypokyphotic AIS patients. Furthermore, Sudo et al. [22] proposed that there was a significant positive correlation between the changes in the TK and the segments of Ponte osteotomes in patients with hypokyphotic thoracic spines. However, some studies have reported a posterior Ponte osteotomy with pedicle screw instrumentation for major thoracic curves did not show a significant improvement in the sagittal correction [23–25]. Though both our study and above researches selected similar Ponte osteotomy method with posterior pedicle screw strategy as the object of research, the surgical outcomes might differ for three reasons. Firstly, there were different inclusion criteria among these studies. Shah et al.[14] and Javier et al. [21] included AIS patients of Lenke types 1–4, another research of Javier et al. [23] included patients with scoliosis of Lenke types 1–6, Halanski et al. [24] included AIS patients of Lenke types 1 and 2, and Takahashi et al. [25] included patients with scoliosis of Lenke types 1, 2, 4, and 6, while we merely enrolled Lenke type 1 AIS patients with a major thoracic curve. Besides, there were various definitions of hypokyphosis. Shah et al. [14] suggested that the hypokyphosis was defined as TK less than 20°, and Sudo et al. [22] suggested that TK less than 15°, while our study and Javier et al. [23] suggested that the hypokyphosis was defined as TK less than 10°. Moreover, the rod materials, rod diameters, and pedicle screw densities were different among the studies. Most of the authors reported that using a 6.0 mm diameter Co–Cr rod and a high pedicle screw density might be considered to optimize the sagittal correction in hypokyphotic patients [26, 27]. In addition, these scholars found that the surgeon who performed the surgery was the most important and only statistically significant predictor for restoring to a normal kyphosis rather than other four preoperative factors, including preoperative TK, rod material, implant density, and whether adoption of Ponte osteotomy [28]. In our study, we not only included the same type of scoliosis patients, but also used identical rod material, rod diameter, and implant density, and applied Ponte osteotomy merely in apex region. Meanwhile, all the surgeries were performed by one single experienced spinal surgeon using the same operative technique. Therefore, we considered that Ponte osteotomy could obtain the contouring of a satisfactory thoracic kyphosis.

Previous literature regarding Ponte osteotomy for coronal correction is conflicting. Most scholars have reported that major thoracic curves of AIS treated with segmental pedicle screw instrumentation and Ponte osteotomies could acquire correction [14, 16, 23, 29]. However, several scholars held opposite opinions [24, 25]. In our study, there was no significant difference in the preoperative main thoracic Cobb angle between the Ponte group and the control group (48.10° ± 3.93° vs. 50.03° ± 4.88°, *P* = 0.056), while the control group presented a larger postoperative main thoracic Cobb angle (20.33° ± 3.75° vs. 15.18° ± 2.84°, *P* < 0.001). The preoperative curve flexibility of AIS patients was similar in two groups. Therefore, we considered that Ponte osteotomy might achieve a posterior tissue release to increase the curve flexibility, so as to improve the coronal main curve correction in AIS patients. To a certain extent, the implants and surgical technology may also affect the coronal correction.

Ponte osteotomy increased the operative time and blood loss in patients with scoliosis. Takahashi et al. [25] reported that the Ponte group displayed a significantly longer surgical time (236 ± 13 vs. 187 ± 9 min, *P* = 0.003) and more blood loss (1141 ± 150 vs. 745 ± 120 ml, *P* = 0.047) for the AIS patients. Halanski et al. [24] also found significant differences in the blood loss per level and the operation time per level between the Ponte group and the control group (97 ± 42 vs. 66 ± 25 ml, *P* = 0.01) (31 ± 5 vs. 23 ± 3 min, *P* < 0.001). Shah et al. [14] reported an average intraoperative blood loss of 1508 ± 874 ml and a mean operative time of 321 ± 63 min in AIS patients who underwent Ponte osteotomies and pedicle screw instrumentation. In our study, the average intraoperative blood loss was significantly higher in the Ponte osteotomy group (1103.25 ± 115.06 vs. 979.75 ± 171.71 ml, *P* < 0.001), as was the operative time (262.00 ± 28.80 vs. 229.50 ± 26.84 min, *P* < 0.001). Though both our study and most researches have reported similar results, the detailed operative time and blood loss were not consistent in different studies. Some researchers explained that the largest amount of intraoperative blood loss mainly occurred during screw insertion [30]. Moreover, we considered that the experience of the surgeons and the number of Ponte osteotomies performed might play a key role in controlling the operation time and blood loss.

The HRQoL was assessed using SRS-22, which has been shown to be a valid and reliable method in assessing the clinical outcome for the surgical treatment of AIS in our country [31]. Shah et al. [14] reported that scores in each domain and total score of SRS-22 were significantly improved after Ponte osteotomies with pedicle screw instrumentation in the treatment of AIS patients. Javier et al. [21] reported that the SRS-22 postoperative scores were similar between the control group and the Ponte group. Takahashi et al. [25] did not find a significant difference between the two groups in each subtotal or total score at one-year postoperatively. In our study, the patients who underwent Ponte osteotomy had lower preoperatively self-image and satisfaction than those in the control group (*P* = 0.020 and *P* = 0.033, respectively). At the 2-year follow-up, we did not find significant difference between the Ponte group and the control group in any domain. These findings were consistent with those reported by Samdani et al. [29] and Aaron et al. [32]. Therefore, though Ponte osteotomy could obtain a better coronal correction and a more desired thoracic kyphosis, these changes were not reflected in the HRQoL.

There were several potential limitations in this study that should be pointed out. Firstly, this was a retrospective study rather than prospective study, and therefore, there may exist data bias and clinical heterogeneity. Secondly, we adopted a relatively short follow-up period in this study which concluded that Ponte osteotomy may obtain better sagittal and coronal alignment in hypokyphotic AIS thoracic patients without significant improvement of HRQoL. With a much longer follow-up, especially if we tracked these patients in their elder stage, the variation of HRQoL might be different. Besides, the population we enrolled were mild thoracic curve AIS patients, and Ponte osteotomy may affect much more in rigid curve patients. What’s more, we adopted full-length lateral X-ray to assess the sagittal profile of the patients in this study, which may not reflect the exact sagittal alignment. Last but not least, the included sample in each group was relatively small. Therefore, a larger-scale, longer follow-up study is necessary to further validate our clinical findings.

## Conclusions

Ponte osteotomy and pedicle screw instrumentation are safe and effective surgical methods for better sagittal contour restoration to treat AIS patients with hypokyphosis of the thoracic segment. However, the application of Ponte osteotomy performed in thoracic curves showed significantly higher blood loss and operative time. Though Ponte osteotomy could improve the coronal correction and obtain the desired thoracic kyphosis in hypokyphotic AIS patients, Ponte osteotomy did not obtain a better HRQoL. Therefore, we considered that the Ponte osteotomy may be an alternative method to restore the desired thoracic kyphosis, which needs further study.

## Data Availability

Access to the data of this study is limited to the research team, study monitors, and the data safety monitoring board. However, upon request, anonymized data can be made available by the principal investigator.
